# First report of junctional epidermolysis bullosa (JEB) in the Italian draft horse

**DOI:** 10.1186/s12917-015-0374-0

**Published:** 2015-03-10

**Authors:** Katia Cappelli, Chiara Brachelente, Fabrizio Passamonti, Alessandro Flati, Maurizio Silvestrelli, Stefano Capomaccio

**Affiliations:** Department of Veterinary Medicine, University of Perugia, Via San Costanzo 4, 06126 Perugia, Italy; Private Practitioner, via Roma 193, Scoppito, L’Aquila, Italy; Institute of Zootechnics, UCSC, via Emilia Parmense 84, 29122 Piacenza, Italy

**Keywords:** Junctional epidermolysis bullosa, Horse, Mechanobullous disease, Electron microscopy, Lamina densa, *LAMC2*, Italian draft horse, Inbreeding

## Abstract

**Background:**

Epitheliogenesis imperfecta in horses was first recognized at the beginning of the 20th century when it was proposed that the disease could have a genetic cause and an autosomal recessive inheritance pattern. Electron microscopy studies confirmed that the lesions were characterized by a defect in the lamina propria and the disease was therefore reclassified as epidermolysis bullosa. Molecular studies targeted two mutations affecting genes involved in dermal–epidermal junction: an insertion in *LAMC2* in Belgians and other draft breeds and one large deletion in *LAMA3* in American Saddlebred.

**Case presentation:**

A mechanobullous disease was suspected in a newborn, Italian draft horse foal, which presented with multifocal to coalescing erosions and ulceration on the distal extremities. Histological examination of skin biopsies revealed a subepidermal cleft formation and transmission electron microscopy demonstrated that the lamina densa of the basement membrane remained attached to the dermis. According to clinical, histological and ultrastructural findings, a diagnosis of junctional epidermolysis bullosa (JEB) was made. Genetic tests confirmed the presence of 1368insC in *LAMC2* in the foal and its relatives.

**Conclusion:**

This is the first report of JEB in Italy. The disease was characterized by typical macroscopic, histologic and ultrastructural findings. Genetic tests confirmed the presence of the 1368insC in *LAMC2* in this case: further investigations are required to assess if the mutation could be present at a low frequency in the Italian draft horse population. Atypical breeding practices are responsible in this case and played a role as odds enhancer for unfavourable alleles. Identification of carriers is fundamental in order to prevent economic loss for the horse industry.

## Background

Junctional epidermolysis bullosa (JEB) belongs to the group of vesiculo-bullous diseases of the epidermis. With this term, several diseases are encompassed that are all characterized by the formation of a split (vesicle or bulla) in any layer of the epidermis or beneath it, at the dermoepidermal junction. This split occurs in two ways: as a consequence of an immune-mediated attack to components of the intercellular and cell-basement membrane adhesion system or as a result of an inherited condition resulting in a lack of any of these components. In this second case, epidermolysis bullosa (EB) is a recessive inherited disease characterized by a genetic defect leading to an inadequate synthesis of structural components of intercellular adhesions such as keratin filaments, desmosomes and hemi-desmosome proteins and anchoring fibrils such as collagen VII [1]. In humans, three subtypes of EB are described that are classified according to the distribution of the lesions and the location of the split in the epidermis and dermis: in simplex EB, the split forms in the basal keratinocyte layer; in junctional EB the split forms in the lamina lucida, leaving the lamina densa anchored to the underlying dermis; in dystrophic EB the split forms within or below the lamina densa which therefore remains attached to the overlying epidermis [2].

Epidermolysis bullosa is recognized in dogs, sheep, horses, cattle, goats and cats [3-7]. Lesions can be present at birth or develop in a short period of time and are characterized by the development of vesicles and bullae that rapidly progress to erosions and ulceration at sites of minor trauma such as the lips, the oral mucosa, the distal extremities and the coronary band, with resulting sloughing of the hoof or claws. Lesions can be secondarily infected and become pustules. Affected animals may die soon after birth due to inability to suckle. Histologically, the lesions show a split that can be intraepidermal, at the dermoepidermal junction or subepidermal. The anatomical location of the split is an important diagnostic criterion because it reflects a different pathogenesis of lesion formation [3].

In horses, two mutations have been associated with the disease, involving two different genes coding for the Laminin 332 protein complex [4,8]. Laminin is a heterotrimeric basement membrane protein integral to the structure and function of the dermal–epidermal junction consisting of three glycoprotein subunits: the **α**3, **β**3 and **γ**2 chains, which are encoded by the *LAMA3*, *LAMB3* and *LAMC2* respectively [9]. A mutation in any of these genes results in the condition known as hereditary junctional epidermolysis bullosa (JEB). An insertion of a cytosine (1368insC) in the *LAMC2* was found in 2002 in draft horses (Belgian Horse, Trait Breton and Trait Comtois) [4,10]. This mutation is responsible for a frame-shift, with consequent premature stop codon formation, leading to a truncated form of the Laminin 332 chain. In 2009, a 6589-bp deletion spanning exons 24 and 27 was found in the *LAMA3* in American Saddlebred foals born with the skin-blistering condition formerly known as epitheliogenesis imperfecta. The deletion confirms that the disease can be classified as JEB and corresponds to Herlitz JEB in humans [8]. In both cases, the inheritance of the disease is a classic Mendelian autosomal recessive.

## Case presentation

**Figure 1:**
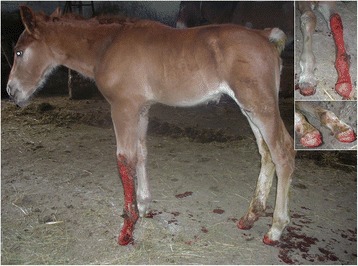
**Italian draft horse foal, male, 4-day-old: Focal extensive, erosive to ulcerative lesions were present in all four legs and particularly severe in the left front leg.** Lesions were covered by crusts and were associated with sloughing of the hoof and bleeding.

**Figure 2:**
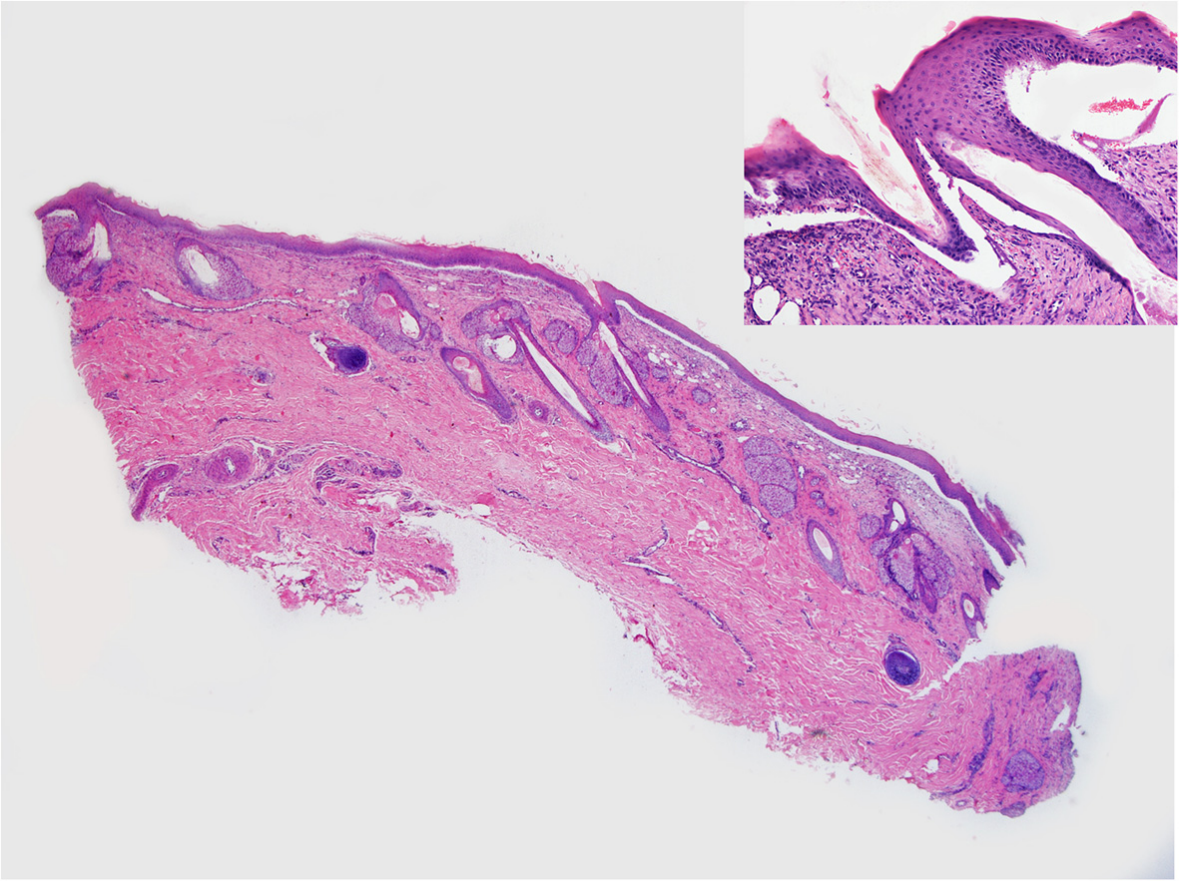
**Italian draft horse foal, male, 6-day-old, biopsy from the coronet: Histological examination showed the presence of a subepidermal cleft with little or no underlying dermal inflammation.** The dermoepidermal separation involved the hair follicle infundibulum as well (insert). HE. 1,25x (insert 10x).

**Figure 3:**
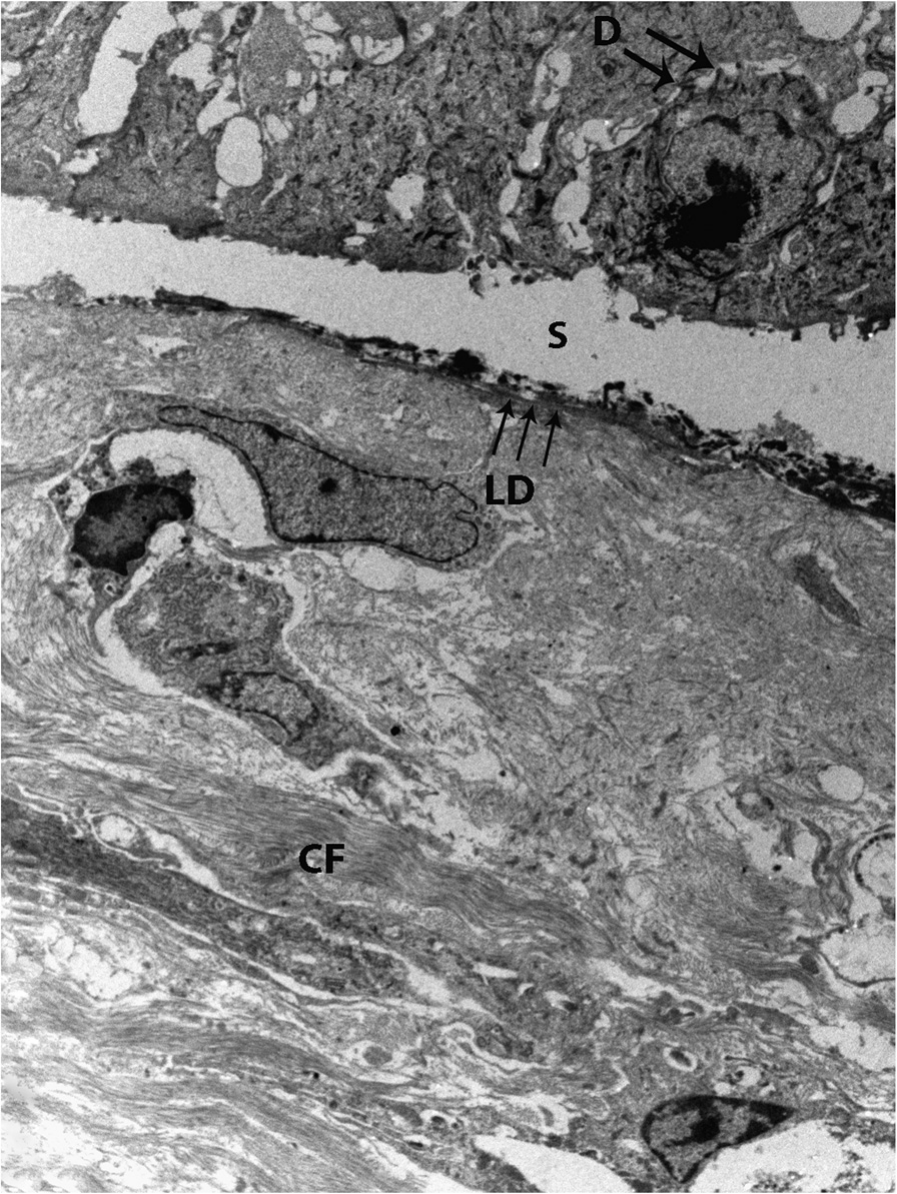
**Transmission electron micrograph of the skin biopsy.** Normal keratinocytes form the roof of the split (S) and the dermis is at its base. Desmosomes (D) are still visible whereas hemidesmosomes are not present. The lamina densa (LD) is located at the base of the split. Normal collagen fibers (CF) are visible in the superficial dermis. TEM. 2,800x.

A male foal coming from a horse farm with 25 animals was born at term with eutocic delivery. The foal, at birth, showed the presence of lesions affecting the distal extremities of all four legs. From the carpus of the left foreleg and from the coronet of the other three legs, the skin was missing and the denuded dermis was covered by debris (Figure 1). No lesions were observed at the mucocutaneous junctions or in oral mucosa. The foal was treated with IV antibiotics (cefquinome 1 mg/kg twice a day and amikacin 15 mg/kg once a day) and a hoof boot was applied to the foot that had lost the hoof. Two days later, four skin biopsies were taken with the owner’s consent from the dorsal and palmar surface of the carpus, from the coronary band and from the coronet at the transition between affected and unaffected areas. Despite treatment, the foal died after 6 days and the owner declined the necropsy. Histologic examination of skin biopsies revealed the presence of vesiculo-bullous lesions characterized by a complete separation of the epidermis from the dermis at the level of the dermoepidermal junction. The blister formation also involved the infundibular portion of hair follicles and ulcerated areas were covered by thick serocellular crusts. Periodic acid-Schiff (PAS) staining revealed a PAS positive lamina densa at the pavement of the blister, attached to the underlying dermis. The macroscopic and histologic lesions were compatible with a hereditary epidermolysis bullosa. According to the PAS staining results, our case was compatible with a junctional epidermolysis bullosa (Figure 2). Transmission electron microscopy from formalin fixed skin biopsies confirmed the presence of a splitting between the epidermis and dermis. Basal keratinocytes were intact and demonstrated normal desmosomes but no hemidesmosomes were identifiable. The lamina densa was present on the pavement, at the dermal side of the blister, consistent with a splitting at the level of the lamina lucida (Figure 3).

**Figure 4:**
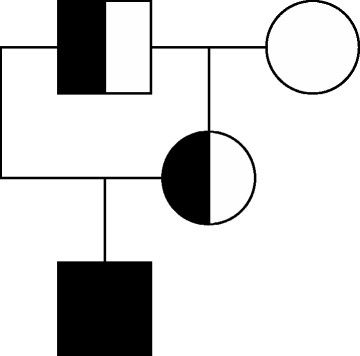
**Pedigree tree of the affected foal, homozygous for the mutation in** ***LAMC2*** **gene (full black square).** Half black figures indicate carrier subjects while open figure equates to the wild type. Squares indicate males while circle female subjects.

Based on the macroscopic and histologic findings as well as the ultrastructural features, a diagnosis of hereditary junctional epidermolysis bullosa was made. Molecular tests aimed at the detection of known mutations associated with the disease, involving the Laminin 5 protein complex, were performed in order to confirm the diagnosis. Nucleic acids were extracted from 200 μl of total blood using the QIAamp DNA Mini Kit (Qiagen) following the manufacturer’s instructions. Since the disease has a Mendelian autosomal recessive inheritance, foal’s DNA together with the DNA of the mother (admixed horse), of the father (heavy horse who was also the grandfather) and of the maternal grandmother (light horse) was tested at the cited loci. PCR was performed as previously described [4,8] using 30 ng of DNA as template for the amplification of *LAMC2* and *LAMA3* regions where the known mutations rely, and amplicons directly sequenced. PCR results were negative for *LAMA3* deletion in all samples. The affected foal was homozygous for 1368insC in *LAMC2* whereas the sire and the dam were heterozygous for the insertion (Figure 4).

These results confirmed that the mutation causing junctional epidermolysis bullosa in the foal was localized in the *LAMC2*, as already described in northern Europe’s coldblood breeds (Belgian Horse, Trait Breton and Trait Comtois) [4,10], which participated, with some lines, to the creation of Italian draft horses [11]; since the disease has a classical autosomal recessive Mendelian inheritance, both parents must be heterozygous (carriers).

Inbreeding, enhanced by erroneous breeding practices, should always be avoided as it can increase the frequency of potentially deleterious recessive alleles in the population and their phenotypic manifestation at individual level [12].

## Conclusion

This is the first report of JEB in Italy. The disease was histologically described as having the typical pathognomonic features and assessed via molecular tests.

Future studies should include genotyping the 1368insC mutation in *LAMC2* in a larger population of Italian Draft horses to determine the allele frequency within this population, and avoid other episodes of JEB.

Identification of carriers is crucial as much as breeder awareness about the avoidance of certain mates in order to prevent economic loss for the horse industry.
